# Health risk assessment of air pollution in Xinjiang, Northwest China

**DOI:** 10.1038/s41598-026-39776-x

**Published:** 2026-02-09

**Authors:** Heping Li, Zhiguo Xue, Bowen Cheng, Yuting Liu, Pengpeng Qin, Yuhan Zhao

**Affiliations:** 1 School of Life and Geography, Kashi University, Kashi, 844000 China; 2 Institute of Desert Meteorology, China Meteorological Administration, Urumqi, 830000 China; 3 Xinjiang Key Laboratory of Desert Meteorology and Sandstorm, Urumqi, 830000 China; 4 Xinjiang Key Laboratory of Tree-Ring Ecology, Urumqi, 830000 China; 5 School of Environment and Health, Jianghan University, Wuhan, 430056 China; 6 College of Atmospheric Sciences, Key Laboratory of Semi-Arid Climate Change, Ministry of Education, Lanzhou University, Lanzhou, 730000 China

**Keywords:** Air pollution, Air quality index, Air health index, Health effect, Environmental sciences, Environmental social sciences

## Abstract

Air pollution disrupts ecological stability, threatens public health, and increases the risk of associated diseases. This study analyzed the spatiotemporal variations of six criteria pollutants from 2015 to 2024 in Xinjiang and their associated health risks using air quality index (AQI), aggregate air quality index (AAQI) and health risk indices (equivalent health risk–based index (EHAQI) and excess risk (ER)). The study was divided into two periods for comparative analysis: period one (2015–2019) and period two (2020–2024). Results showed that PM₁₀ dominated as the primary pollutant, particularly in Southern Xinjiang. Single-pollutant metrics underestimated combined health impacts of the six pollutants. Environmental policies and lockdowns significantly reduced SO₂ (47.9%), CO (41.7%), PM₂.₅ (19.9%), NO₂ (16.4%), and PM₁₀ (12.0%) compared with Period one. The O₃ growth rate in Northern Xinjiang exceeded that in Southern Xinjiang by 3.7%. Health risk assessments revealed that PM₁₀-driven excess risk (ER) accounted for 80% of the total health burdens in Southern Xinjiang. While in Northern Xinjiang, NO₂ dominated ER, surpassing PM_10_. Spring and winter saw peak EHAQI values, and 36.0% of residents were exposed to “severe” pollution in spring. These findings could provide scientific evidence for PM reduction policies in Southern Xinjiang and pollution-related disease prevention.

**Supplementary Information:**

The online version contains supplementary material available at 10.1038/s41598-026-39776-x.

## Introduction

Air pollution, a critical global environmental and public health challenge, has posed persistent threats to ecosystems and human health due to its complex chemical composition and transboundary transport characteristics. The World Health Organization (WHO) global air quality guidelines 2021 identified particulate matters (PM_2.5_/PM_10_), O₃, and nitrogen oxides (NO_x_) as priority pollutants, emphasizing the necessity of implementing multi-pollutant collaborative control strategies^1,2^. As the largest developing country, China has promoted the pollutant reduction through national strategies such as the “Blue Sky Defense War,” achieving significant progress in controlling traditional pollutants like sulfur dioxide (SO₂) and carbon monoxide (CO)^3^. However, frequent dust storms and worsening O_3_ pollution remain urgent issues, particularly in arid northwestern regions^4^. Featuring the unique topography of “three mountains enclosing two basins”, Xinjiang is surrounded by the Taklamakan Desert (TD), a major natural dust source. With an annual average of 15–20 dust storms, PM_10_ concentrations in the region remain persistently high, which has become a key bottleneck for regional air quality improvement^5,6^.

Arid and semi-arid regions worldwide faced compound pollution from dust aerosols and anthropogenic emissions internationally^7,8^. Studies in the southwestern United States, the middle east, and central Asia showed that mineral catalysis on dust particle surfaces could significantly enhance O_3_ formation and secondary pollutant transformation^9^. Air pollution in China differed significantly across regions. PM_2.5_-O_3_ co-pollution was dominant in central and eastern regions (such as the Fenwei Plain), whereas PM pollution prevailed in northwestern regions (e.g., Xinjiang and Gansu provinces), driven by natural and anthropogenic sources^10,11^. The pollution causes of Xinjiang integrated natural attributes, human activities, and short-term perturbations from public health events. The Taklamakan Desert (TD) releases massive amounts of dust into its surrounding areas each year, which through atmospheric circulation, affects most regions in Southern Xinjiang^12^. Coal consumption and traffic emissions in Northern Xinjiang industrial cities (such as Urumqi and Karamay), and fertilizer volatilization and straw burning in agriculture in Southern regions, collectively led to the uneven regional distribution of pollutant^13,14^. During the COVID-19 lockdowns, global industrial shutdowns and traffic restrictions reduced NO and PM emissions by 20%–30%, while O_3_ increased due to weakened NOₓ transformation, demonstrating the significant impact of anthropogenic policy interventions on pollutant emissions^15,16^.

Traditional single-pollutant evaluation systems, such as air quality index (AQI), underestimated health risks due to neglecting synergistic effects of multiple pollutants^17^. For example, combined exposure to PM_10_ and O₃ could increase the risk of respiratory diseases by over 30%^18^. Comprehensive assessment tools addressed this limitation: the accumulated risk quality index (AAQI) quantified the superposition effect of multiple pollutants through concentration weighting^19^. For instance, the synergistic effect of PM_10_ and NO₂ during the heating season increased “very unhealthy” days by 22%^20^. A study that comprehensively assessed the impact of changes in pollutants on social factors in China found that there were significant differences in health risk index (HAQI) in the central and eastern regions, and the population-standardized HAQI further reflected this inequality^21^.The health hazards of air pollutants were widespread, and triggered complex pathological processes through multi-mechanism synergies. PM caused physical deposition in the respiratory tract, and its mineral components catalyzed the production of reactive oxygen species (ROS), leading to lipid peroxidation and DNA damage. Gaseous pollutants such as NO₂ and O₃ activated the NF-κB inflammatory pathway, inducing the release of pro-inflammatory cytokines and pulmonary fibrosis. Long-term exposure to both could synergistically promote gene mutations and cardiovascular risks^22,23^. Accurately assessing compound pollution risks in arid regions was of great and special significance.

Air pollution not posed independently threats to human health, but could act synergistically with social factors (e.g., population and gross domestic product) and environmental factors (e.g., surface characteristics and meteorological characteristics) to generate combined health risks^23–25^. These factors collectively led to regional disparities in outcomes and imbalances in the degree of exposure inequality^25^. Recent evidence from a national epidemiological investigation in China, indicated that air pollutants (e.g., NO_2_, O_3_, and oxidative potential) could interact with arid conditions and significantly magnify cardiovascular health risks^26^. Xinjiang, especially the southern region, featured a typical continental arid climate with severe dust pollution, thereby imposing substantially greater health risks on the local residents. Previous studies mostly adopted single health indices for selected pollutants, and their study areas were largely concentrated in developed cities in eastern China^10,11,18^. Xinjiang, particularly its southern regions, featured a typical continental arid climate with frequent dust events and severe complex mixed pollution^5^, which posed substantial health risks to local residents. Therefore, comparative investigations into the health risks driven by multiple pollution indices and multi-factor influences under arid backgrounds in Xinjiang were of great practical significance.

In this study, daily observational data for six conventional air pollutants (PM_10_, PM_2.5_, O₃, NO₂, SO₂, CO) were collected in Xinjiang during 2015–2024. Employing diverse air quality discriminant indices, we examined the spatiotemporal variations and regional inequalities of ambient air quality. This study innovatively explored follow aspects: firstly, it conducted a long-term, multi-index health risk assessment in the arid, PM- and NO₂- dominated environment; secondly, it presented a spatio-temporal comparison of health risks driven by different pollutants across Xinjiang; finally, it explored the implications of multi-pollutant exposure under extreme climatic and environmental conditions. The findings of this study could provide a scientific basis for relevant authorities in Xinjiang to formulate targeted environmental policies and for local residents to take evidence-based seasonal preventive measures.

## Method

### Study region

The Xinjiang Uygur Autonomous Region (Xinjiang for short) is situated in the northwestern frontier of China, at the confluence of the Tianshan mountains in central Asia, the arid Tarim Basin, and the Pamir Plateau. It is the largest provincial-level administrative region in China. Since ancient times, Xinjiang has served as a crucial corridor for political, economic, cultural, and civilizational exchanges between the East and the West across the Eurasian continent. The typical temperate continental arid climate results in higher precipitation in mountainous areas and lower precipitation in the plains, with overall high evaporation rates. The average precipitation across the entire region was only 196.2 mm in 2024^5,27^.

**Fig. 1:**
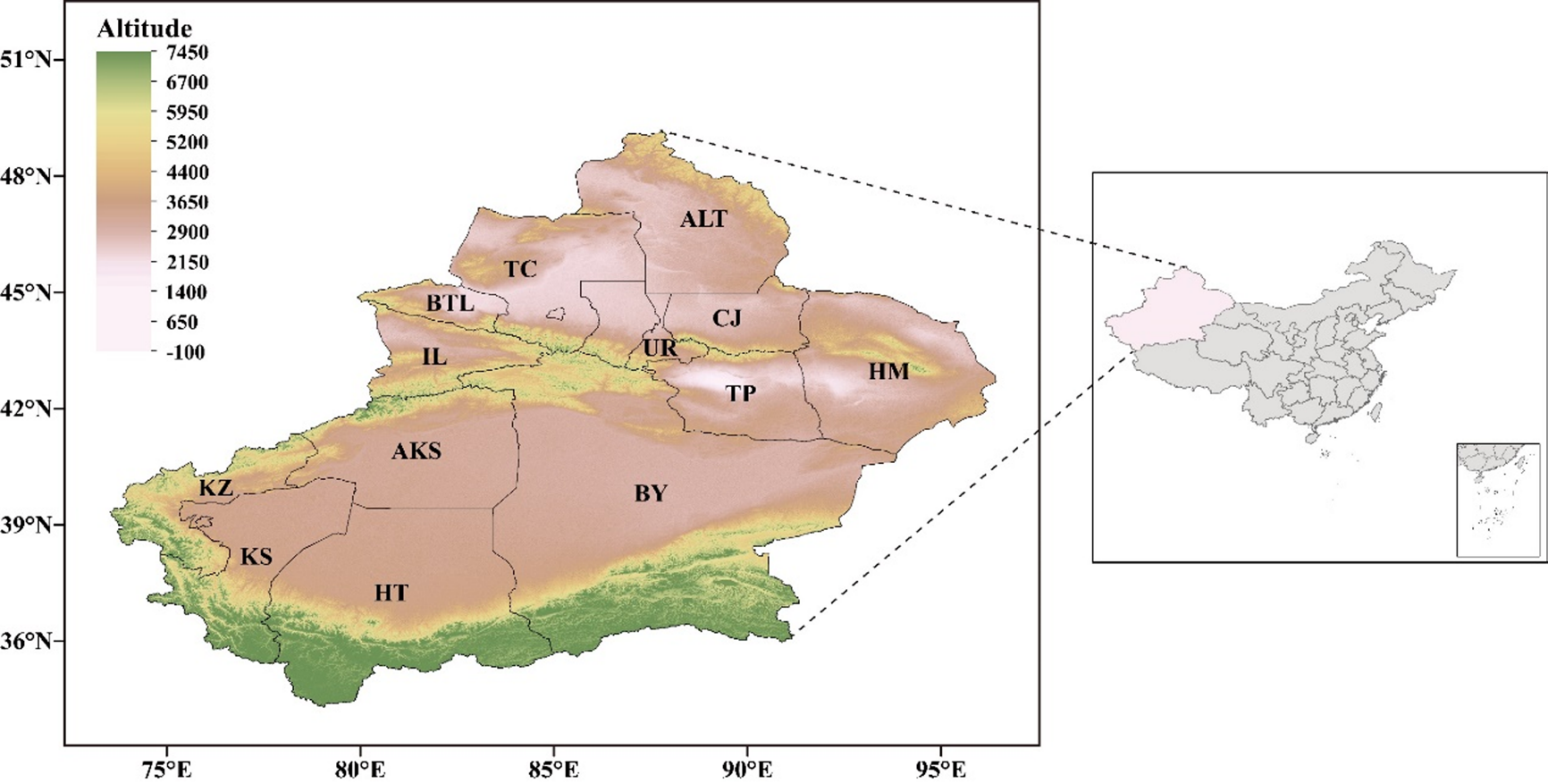
Map of Xinjiang Uygur Autonomous Region.

Based on the administrative divisions of Xinjiang (Fig. 1), the specific study area (abbreviations in parentheses) includes: Northern Xinjiang: Altay (ATL), Tacheng (TC), Bortala (BTL), Ili (IL), Changji (CJ), Urumqi (UR), Turpan (TP), Hami (HM); Southern Xinjiang: Aksu (AKS), Kashgar (KS), Hotan (HT), Bayingol (BY), Kizilsu (KZ).

### Data sources

Daily concentrations of PM_10_, PM_2.5_, CO, NO_2_, and SO_2_, along with 8-hour average O_3_ levels for 13 prefectures in Xinjiang between January 1, 2015, and December 31, 2024, were obtained from the China National Environmental Monitoring Center. The pollutant data for each prefecture represent the average concentrations from monitoring stations within its jurisdiction. Considering the impacts of relevant environmental policies and public health emergencies due to COVID-19^28^, the study was divided into two periods for comparative analysis: period-one (2015–2019) and period-two (2020–2024). The population and mortality rate data for all regions of Xinjiang were all sourced from the Statistical Yearbook of Xinjiang Province (https://tjj.xinjiang.gov.cn/tjj/zhhvgh/list_nj1.shtml).

### Air quality index (AQI)

The Air Quality Index (AQI) has been widely adopted as a composite indicator for assessing air pollution levels. In this study, we employed the official AQI calculation methodology issued by China’s Ministry of Environmental Protection (MEP) to ensure consistency with national standards. The sub-index for each pollutant (AQI_*i*_) is calculated using a piecewise linear interpolation method (Eq. 1a and 1b):

1a,1b $$\:{\mathrm{AQI}}_{i}=\left\{\begin{array}{cc}\frac{{\mathrm{AQI}}_{i,j}-{\mathrm{AQI}}_{i,j-1}}{{C}_{i,j}-{C}_{i,j-1}}\times\:\left({C}_{i}^{obs}-{C}_{i,j-1}\right)+{\mathrm{AQI}}_{i,j-1},&\:j>1\\\:{\mathrm{AQI}}_{i,1}\times\:\frac{{C}_{i}^{obs}}{{C}_{i,1}},&\:j=1\end{array}\right.\:$$

where $\:i$ denotes the pollutant, $\:j$ represents the health category threshold, $\:{C}_{i}^{obs}$ is the measured concentration of pollutant $\:i$, and $\:{C}_{i,j}$ and $\:{\mathrm{AQI}}_{i,j}$ correspond to the concentration and AQI values at the $\:j$-th threshold, respectively. The overall AQI is determined as the maximum sub-index value:

2 $$\:\mathrm{AQI=max}\left\{{\mathrm{AQI}}_{1},{\mathrm{AQI}}_{2},\dots\:,{\mathrm{AQI}}_{n}\right\}\:\left(n=1,2,\dots\:,6\right)\:$$

### Aggregate air quality index (AAQI)

To comprehensively assess the combined effects of multiple pollutants, the aggregate air quality index (AAQI) is defined as^19^:

3 $$\:\mathrm{AAQI=}{\left(\sum\:_{i=1}^{n}{\left({\mathrm{AQI}}_{i}\right)}^{\rho\:}\right)}^{\frac{1}{\rho\:}}\:$$

The sub-index AQI_*i*_ represents the AQI for pollutant *i*, where *ρ* denotes an empirical constant. The pollutant-independent constant ρ is a key parameter in the calculation of the Ambient Air Quality Index (AAQI). Previous studies had suggested that the value of ρ should range between 2 and 3^29,31^, though the most appropriate value for ρ had remained controversial. An earlier study had examined the impact of ρ on AAQI results by adopting different values of ρ (2, 2.5, and 3.0). It had shown that the selection of ρ did exert a limited influence on the computed AAQI values, yet the AAQI/AQI ratio had varied little across the study subgroups^18^. Similar sensitivity experiments were also conducted in Appendix 1, as detailed in Tabel S1. Based on the above analyses, we finally set the parameter ρ to 2. To enable direct comparison with the AQI framework, the AAQI employs identical health categories as the AQI and shares the 0–500 scale range.

### Health risk-based indices

#### Relative risk and excess risk

Human health risks can be quantitatively linked to the magnitude of divergence between observed pollutant concentrations and regulatory standards. This methodology has been extensively applied in environmental epidemiology, utilizing excess risk (ER) and relative risk (RR) coefficients to assess health threats posed by specific pollutants^29^. The *RR*_*i*_ for pollutant *i* is calculated as:

4 $$\:{RR}_{i}={exp}\left[{\beta\:}_{i}({C}_{i}^{obs}-{C}_{i}^{std})\right],{C}_{i}^{obs}>{C}_{i}^{std}$$

The total ER is the sum of individual ER values:

5 $$\:{\mathrm{ER}}_{\mathrm{sum}}=\sum\:_{i=1}^{n}\left(R{R}_{i}-1\right)\:$$

where *β*_*i*_ represents the exposure–response coefficient (health risk per unit increase of pollutant i), *C*_*i*_^*obs*^ is the measured concentration, and *C*_*i*_^*std*^ denotes the risk threshold. The *β* values were derived from a meta-analysis of Chinese epidemiological studies^30^, with the 0.032%, 0.038%, 0.13%, 0.081%, 0.048% and 3.7% for PM_10_, PM_2.5_, NO_2_, SO_2_ and O_3_, respectively. Here, *C*_*i*_^*std*^ corresponds to the Chinese ambient air quality standards (CAAQS) Grade II limit, with concentrations below this level assumed to pose no excess health risk.

#### Equivalent concentration and EHAQI

The Health-risk based AQI (HAQI) was developed to better quantify the association between air pollutants and population health risks^23^. Existing studies have conventionally employed equivalent concentration *C*_*i*_^*equ*^ to calculate the HAQI, aligning its scale limits with the AQI for comparative analysis. Here, *C*_*i*_^*equ*^ represents the concentration of pollutant *i* at which its excess risk (ER*i*) equals the sum of population risk (ER_*sum*_). While the direct application of *C*_*i*_^*equ*^ may lead to substantial overestimation of pollutant-induced health risks, necessitating the implementation of threshold-dependent partitioning in methodological refinement^18^. The specific optimized equivalent concentration calculation framework as follows:

6a,6b $$\:{C}_{i}^{equ}=\left\{\begin{array}{cc}\frac{ln\left(\mathrm{E}{\mathrm{R}}_{\mathrm{sum}}+1\right)}{{\beta\:}_{i}}+{C}_{i}^{std},&\:{C}_{i}^{obs}>{C}_{i}^{std}\\\:{C}_{i}^{obs},&\:{C}_{i}^{obs}\le\:{C}_{i}^{std},\end{array}\:\:\:\:\:\:\:\:\right.$$

Building upon the established framework for equivalent concentrations and AQI calculations, the index equipped with health-risk and contamination level were quantified through the equivalent health risk–based index (EHAQI)^31^, derived analogously to conventional AQI using the following partitioned functions (Eq. 7a, 7b):

7a,7b $$\:{\mathrm{EH}\mathrm{AQI}}_{i}=\left\{\begin{array}{cc}\frac{{\mathrm{AQI}}_{i,j}-{\mathrm{AQI}}_{i,j-1}}{{C}_{i,j}-{C}_{i,j-1}}\times\:\left({C}_{i}^{equ}-{C}_{i,j-1}\right)+{\mathrm{AQI}}_{i,j-1},&\:j>1\\\:{\mathrm{AQI}}_{i,1}\times\:\frac{{C}_{i}^{equ}}{{C}_{i,1}},&\:j=1\end{array}\right.\:$$

The overall EHAQI is determined as:

8 $$\:\mathrm{EH}\mathrm{AQI=max}\left\{{\mathrm{EHAQI}}_{1},{\mathrm{EHAQI}}_{2},\dots\:,{\mathrm{EHAQI}}_{n}\right\}\:\left(n=1,2,\dots\:,6\right)\:$$

### Mortality burden Estimation

The additional mortality attributable to air pollutant is estimated using^31^:

9 $$\:\varDelta\:Y=\:Mortalit{\mathrm{y}}_{0}\cdot\:ER\cdot\:\mathrm{Population}$$

Where, *ΔY* denote pollution-attributable excess deaths, ER is the mortality rate percentage change from air pollution exposure, *Population* means the exposure population to pollutants, and *Mortality*_*0*_ is the baseline total mortality rate. A substantial disruption in mortality trends occurred between 2020 and 2024 due to COVID-19 pandemic effects. Consequently, the 2015–2019 average mortality risk (*Mortality*_*0*_) was utilized as the baseline (Period-two) to ensure temporal consistency in risk attribution.

## Results

**Fig. 2:**
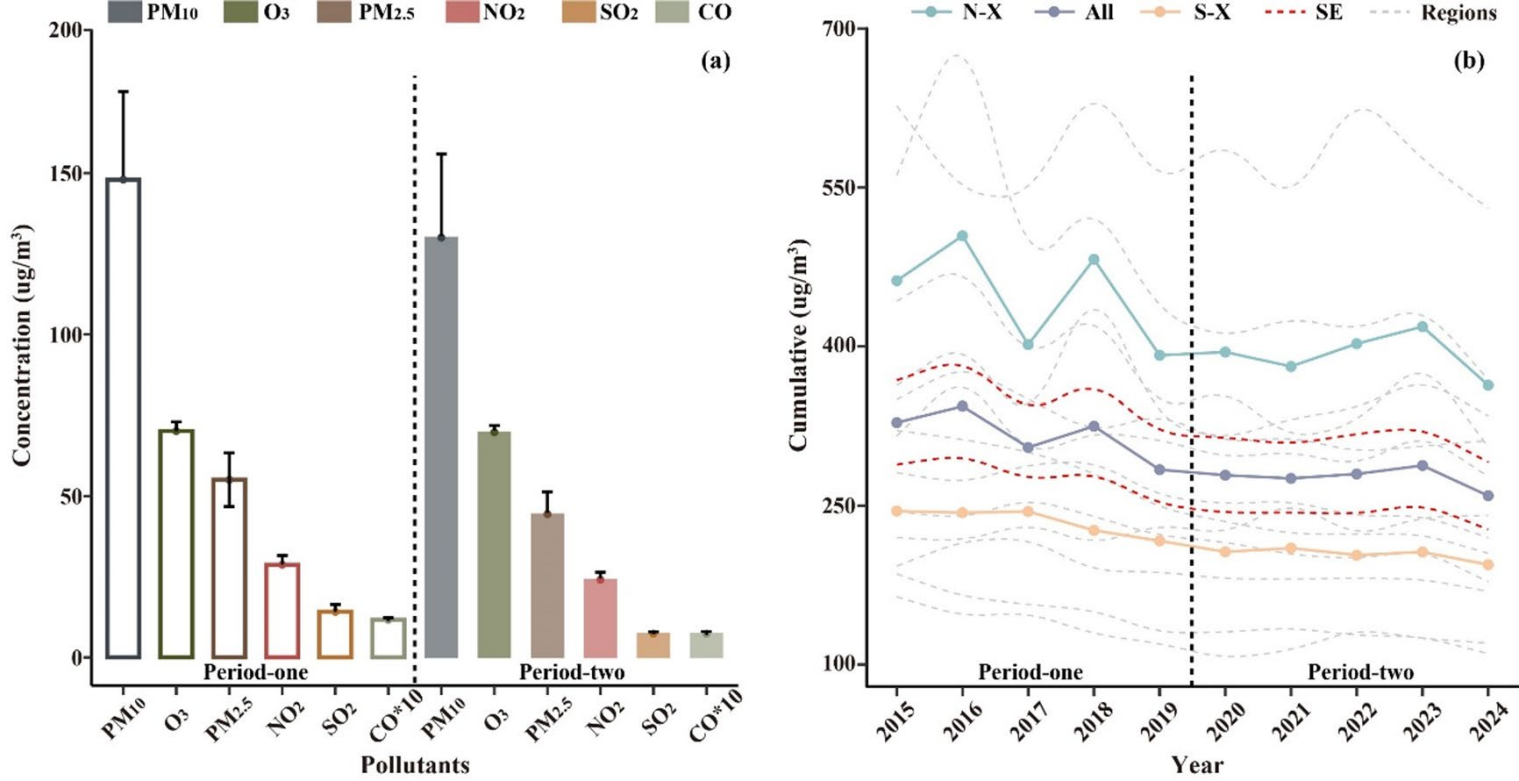
Comparative analysis of pollutant concentrations between the Period one and two in Xinjiang (a), along with the temporal trends of regional cumulative concentrations from 2015 to 2024 (b). (N-X: Northern Xinjiang, S-X: Southern Xinjiang).

Comparative analysis of pollutant concentrations between the Period one and two in Xinjiang, along with the temporal trends of regional cumulative concentrations from 2015 to 2024 were shown in Table S2 and Fig. 2a. The atmospheric pollution in Xinjiang was predominantly characterized by particulate matter (PM), with the highest annual mean concentration of PM_10_ (139.3 µg/m^3^) and then PM_2.5_ (49.7 µg/m^3^). Southern Xinjiang generally demonstrated higher concentrations of all pollutants than Northern Xinjiang, with PM exhibiting the most pronounced disparity (nearly threefold difference). Among Northern Xinjiang cities, Turpan showed the most severe pollution levels, while Hotan was the most polluted in Southern Xinjiang. Pollutant concentrations exhibited a significant decreasing trend, with average reduction magnitudes during Period two (relative to Period one) descending in the following order of SO_2_: 47.9%, CO: 41.7%, PM_2.5_: 19.9%, NO_2_: 16.4% and PM_10_: 12.0% compared, except for O₃ concentrations showed a marked 9.9% increase on the contrary. Cumulative concentration folds generally demonstrated a consistent annual decreasing trend (Fig. 2b), with an average decline rate of 2.3% per year. Regional variations were observed: Southern Xinjiang showed a 1.8% annual reduction, while Northern Xinjiang demonstrated a 2.5% decrease. Kashgar displayed the most significant reduction among all regions, averaging − 2.8% per year. Uniform units (µg/m³) were used for comparison to avoid distorting cumulative values. Throughout the study periods, only the inter-annual mean concentrations of PM_10_ and PM_2.5_ consistently exceeded the CAAQS Grade II, while other pollutants remained below the regulatory limits. The average standard error (SE) from 2015 to 2024 was ± 36.6 µg/m³.

**Fig. 3:**
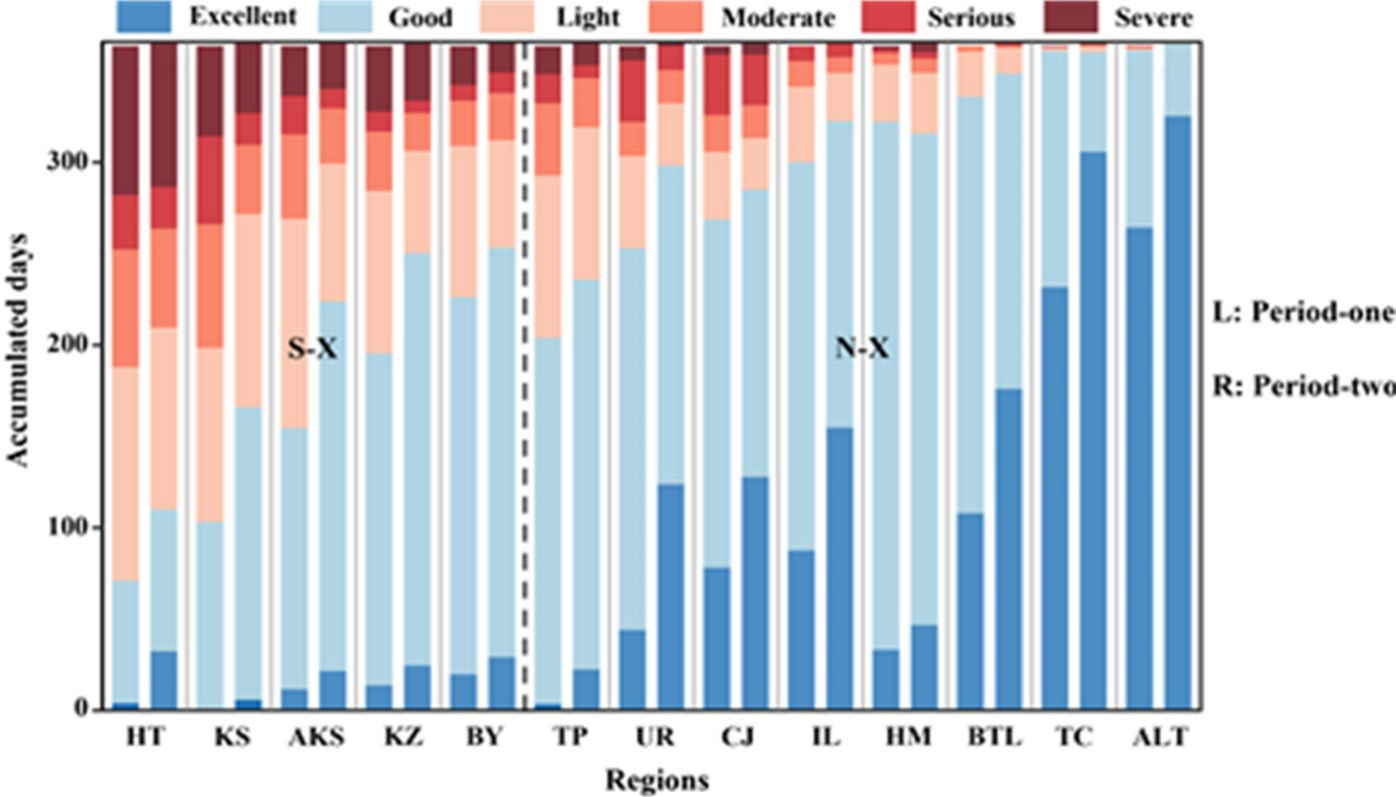
Annual mean cumulative days of each AQI types (six) in 13 regions of Xinjiang during the two periods.

The annual average cumulative days of each of the six AQI types in 13 regions of Xinjiang during different periods were shown in Fig. 3. From Period one to two, notable improvements emerged in pollution patterns: The proportion (relative to one year) of days on light pollution and worse categories dropped from 33.2% to 25.8%, with moderate pollution days experiencing the steepest decline (−26.9%). Conversely, Good and better days rose from 66.6% to 74.5%, driven by a 55% surge in Excellent days. Regarding pollution improvement rates, Aksu recorded the most substantial reduction in Severe pollution (−32.3%), whereas Urumqi achieved the highest growth in Excellent days (181.4%). Comparing regional disparities, Southern Xinjiang regions consistently exhibited higher pollution levels, with 51.9% of annual days classified as Light pollution or worse. Hotan emerged as the most polluted area, accounting for 75.1% of days exceeding the acceptable thresholds. In contrast, Northern Xinjiang regions maintained superior air quality, with all areas surpassing 60% on Good and Excellent days. Altay led with 99.5% of days meeting this criterion. Averagely, the polluted days in Southern Xinjiang (288 days) was 3.42 times that in Northern Xinjiang (55 days).

**Fig. 4:**
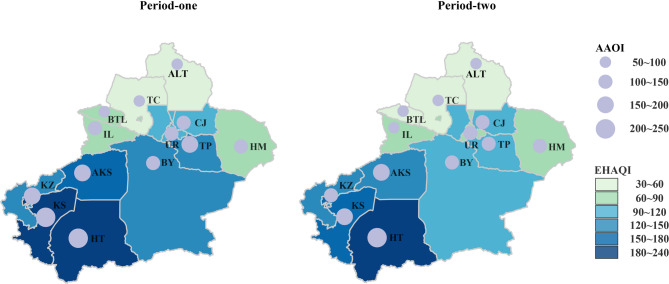
Spatial variation in annual mean AAQI and EHAQI in 13 regions of Xinjiang.

The spatial distributions of Xinjiang’s annual average AAQI and EHAQI across different periods were depicted in Fig. 4. Both indices spatially demonstrated the general characteristics of “Higher in Southern and lower in Northern”, decreasing sequentially from south to north, and being notably lower in the second stage than in the first stage. Due to high-concentration pollution, both AAQI and EHAQI remained generally high in Southern Xinjiang. Specifically, Hotan exhibited the highest values, followed by Kashgar. The average AAQI and EHAQI in Hotan exceeded 233.5/year and 223.7/year, respectively. In Northern Xinjiang, AAQI and EHAQI indices remained at low levels, with Altay showing the lowest values, followed by Tacheng. The average AAQI and EHAQI in Altay were lower than 56.2/year and 41.7/year, respectively. From Period one to Period two, in Kashgar of Southern Xinjiang, the EHAQI demonstrated the greatest reduction (−24.5%), and AAQI decreased by −18.3%. In Urumqi of Northern Xinjiang, the largest reduction of 32.0% for EHAQI and a 26.3% decrease in AAQI were observed.

**Fig. 5:**
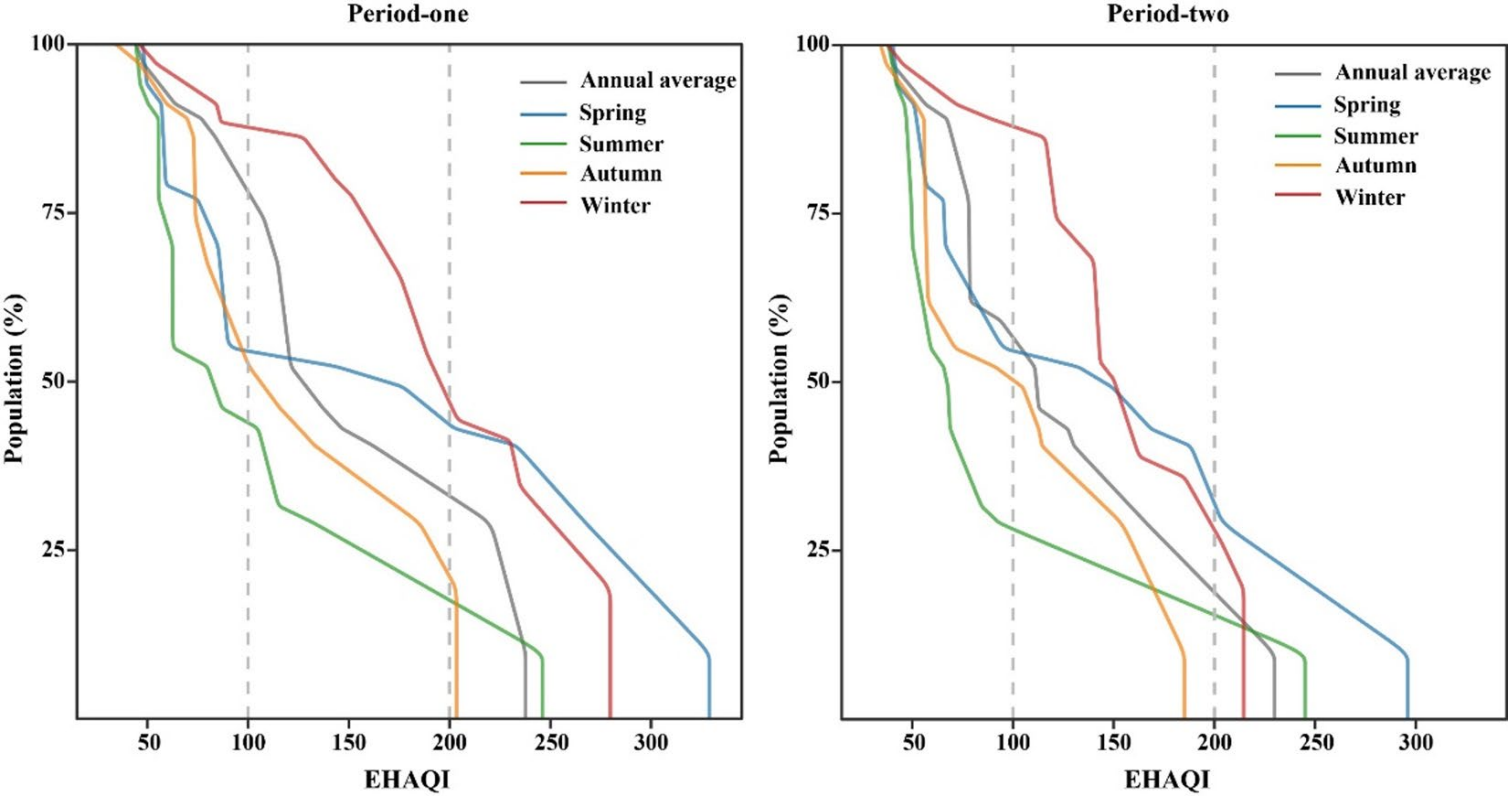
ER values and proportions of each pollutant in the two periods.

The analysis of excess mortality risk (ER) caused by each pollutant across different periods was shown in Fig. 5. The overall ER was dominated by the effects of PM particulates, with annual average value of 2.4%. While the mortality effects of NO₂ also warranted attention, with its ER value ranking third. In some Northern Xinjiang cities, the annual average ER of NO₂ even exceeded that of PM, such as Urumqi (2.3%) and Changji (1.9%). On account of the generally higher pollution levels in Southern Xinjiang, the total ER values (10.1%) were significantly higher than those in Northern Xinjiang (3.4%). Hotan exhibited the highest total ER across all regions (15.5%), while Altay had the lowest (0.4%). In Period two, ER values in all regions decreased. The average reduction in total ER in Northern Xinjiang (35.9%) was greater than that in Southern Xinjiang (22.5%), with the largest decline observed in Altay (75.3%). In the analysis of ER attribution proportions, the percentage of ER attributed to PM_10_ increased by 5.6%, while the proportions attributed to other pollutants decreased.

**Fig. 6:**
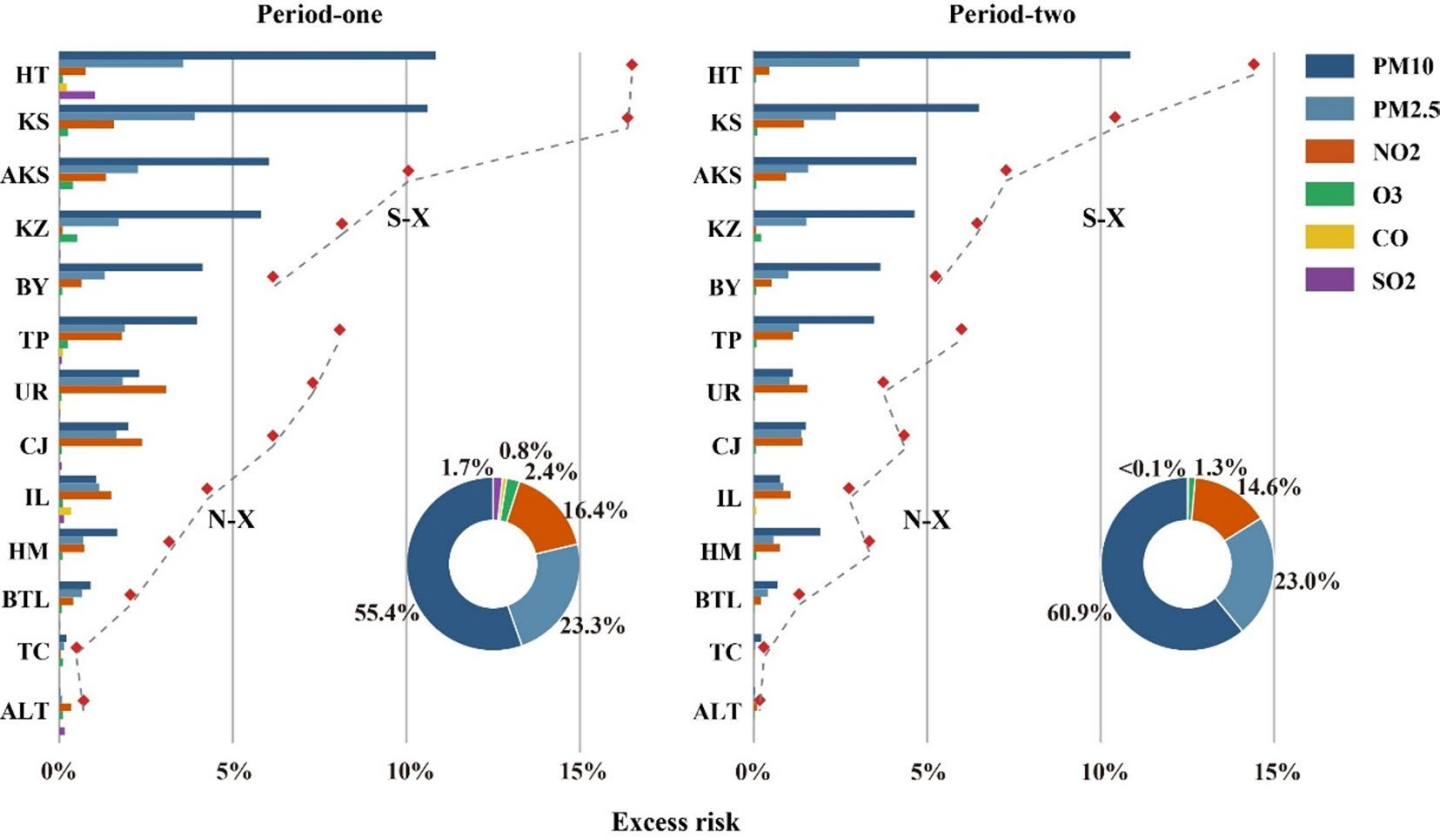
Cumulative distribution of population-weighted estimations for average EHAQI in Xinjiang in the two periods.

The population statistical data of various regions in Xinjiang were integrated with EHAQI indices to determine the population-weighted cumulative distributions during the two Periods, thereby more accurately quantifying the proportion of the population exposed to air pollutants, as shown in Fig. 6. During Period-one, 74.2% of Xinjiang’s population was exposed to polluted air (EHAQI > 100), and 29.0% were exposed to Severe pollution or worse (EHAQI > 200). The severity of air pollution varied by season, being most severe in spring and winter. In spring, 43.0% of the population was affected by Severe pollution or worse, while this proportion reached 44.3% in winter. In contrast, air quality was relatively better in summer and autumn, with 9.7% and 19.3% of the population affected by severe pollution or worse, respectively. In Period-two, air quality improved significantly compared to Period-one, with the population living under better air quality (EHAQI < 100) increasing to 47.8%. The proportions of polluted populations decreased notably in summer and autumn. However, an average of 27.6% of the population remained exposed to Severe pollution in spring and winter. Regional disparities also existed: in almost all seasonal patterns, such as residents in Hotan and Kashgar of Southern Xinjiang were always exposed to the highest levels of EHAQI, while populations in Altay and Tacheng of Northern Xinjiang consistently experienced low levels.

**Fig. 7:**
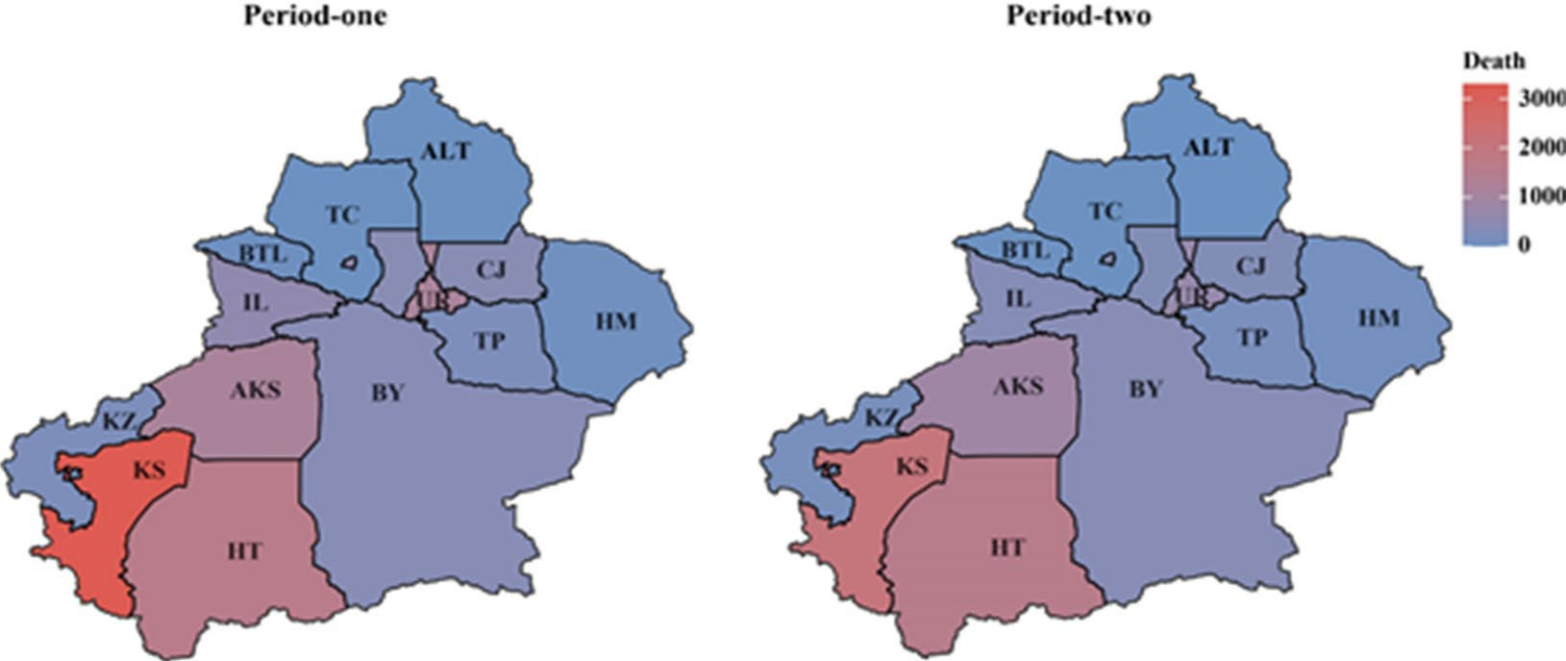
Spatiotemporal variations in annual average mortality attributed to air pollution in Xinjiang.

The spatiotemporal variations in annual average mortality attributed to air pollution in Xinjiang were presented in Fig. 7. Similar to the spatial distribution of pollution patterns, the assessed mortality values exhibited a gradient of higher in the Southern and lower in the Northern, alongside a decreasing trend over time. For Period one, there were 706 annual average deaths due to air pollution. Owing to severe air pollution and higher population density, Kashgar recorded the highest annual average mortality from air pollution (3,270 deaths). In Period two, the province-wide annual average deaths attributed to air pollution decreased to 522, representing a 26.1% reduction. However, regional disparities persisted. In Northern Xinjiang, all regions except Hami experienced mortality decreases, with an average reduction of 33.9%. Urumqi achieved the largest decline (41.6%) among these. In Southern Xinjiang, the average reduction in deaths reached 23.1%, while Kashgar leading the region by achieving a 36.3% reduction—the most substantial decline of Southern.

## Discussion

In this study, we statistically analyzed the spatiotemporal variations of six typical pollutants in Xinjiang from 2015 to 2024 and evaluated related health risks. The overall characteristics showed that pollution patterns and associated health risks in Southern Xinjiang were more severe than those in Northern Xinjiang, though this situation had improved to varying degrees over time across regions. The specific pollution distribution was influenced by multiple factors in Xinjiang, such as geography, society, and the environment. While the improvements were attributed to factors such as artificial modification of the underlying surface, emission reductions due to regulatory measures, and changes in meteorological conditions.

Particulate matters especially PM₁₀, became the dominant factor affecting the atmospheric environment in Xinjiang. Its contribution ratio to pollution sources in Southern Xinjiang was higher than that in Northern Xinjiang. Many studies have confirmed that dust emitted from natural sources was an important source of PM^32,33–35^. The northwest region of China was affected by three major dust source areas: Mongolia, the gobi desert, and the taklamakan desert^36^. Among them, the Taklamakan Desert was the main source of dust in Xinjiang^37^, while dust sources and wind force were important conditions for the occurrence of sandstorms in this region^38^, which was also a key factor contributing to the significantly higher PM levels in Southern Xinjiang than in Northern Xinjiang. Hotan and Kashgar stood as the highest PM concentrations in Southern Xinjiang, situated in its southwestern expanse. The increase of southwester speed urged areas including Hotan and Kashgar key hotspots for pollutant accumulation^39^. This dynamic likely serves as a key factor underlying the significantly elevated PM levels in Southern Xinjiang compared with other regions.In the recent period, PM level in Xinjiang had decreased remarkably. This might have been related to the sustainable development policies implemented in China since the 20th century, such as the construction of green sand-prevention belts and afforestation. These measures had changed surface conditions such as land types and vegetation status, enhancing the effect of windbreak and sand-fixation^40^. In addition, meteorological factors affecting the frequency of sandstorms also included average wind speed, precipitation, etc^41^.

The concentration of O₃ in Southern Xinjiang was significantly higher than that in Northern Xinjiang, primarily due to two key factors. Initially, the Taklamakan Desert in Southern Xinjiang served as a major natural dust source, and the mineral components in dust (e.g., montmorillonite and kaolinite) could catalytically promote O₃ formation through heterogeneous reactions^42^. Subsequently, the high temperatures and low humidity in Southern Xinjiang accelerated photochemical reactions, providing favorable conditions for O₃ production^9^. In contrast, the faster growth rate of O₃ in Northern Xinjiang (13.6%) compared to Southern Xinjiang (9.9%) could be attributed to follow reasons. First, the reduction of nitrogen oxides (NO_x_) in Northern Xinjiang, caused by the implementation of desulfurization and denitrification technologies in coal-fired power plants, might lead to a decrease in the titration effect of NO on O₃, thereby increasing O₃ levels^43^. Second, the decline of PM concentrations weakened the scattering effect, enhancing photolysis rates and promoting O₃ generation^44^. Finally, the rise in average temperature and the decrease in wind speed might also provided favorable meteorological conditions for ozone accumulation^45^. Regarding the improvement of other pollutants, SO₂ and CO showed the most significant reductions, with average decreases of 47.9% and 41.7%, respectively. Which might have been related to the implementation of desulfurization price subsidy policies, which promoted the popularization of clean energy technologies, as well as the improvement of motor vehicle emission standards^46^.

During the COVID-19 pandemic (2019–2023), strict lockdown measures in China significantly reduced anthropogenic emissions of major air pollutants (excluding O₃) across the country^47–49^. Evidence from national and regional studies highlights distinct patterns of pollution reduction and underlying mechanisms: a study based on near-real-time activity data found that during the COVID-19 restrictions, emissions of PM_2.5_, SO_2_, CO and NO_x_ decreased by 24%, 27%, 28% and 36%, respectively, due to the reduction in emissions from the transportation and industrial sectors^48^. In Xinjiang, the annual average concentration of PM_10_ in 14 prefectures and autonomous prefectures decreased by 4.0% year-on-year, and the annual average concentrations of SO_2_, NO_2_, and CO all decreased by 11.1% year-on-year in 2020^50^. These evidences demonstrated that strict pandemic control measures effectively mitigated anthropogenic pollution in Xinjiang, highlighting the critical role of policy interventions in improving air quality^51,52^.

The singular analysis of individual pollutant indicators could lead to failure in considering the combined effects of multiple pollutants, while the AAQI and EHAQI have been widely adopted as indices for assessing comprehensive pollution and health impacts^31,53^. Hu et al. systematically compared AQI, AAQI, and HAQI across six Chinese cities, revealing that traditional AQI systematically underestimated health risks under multi-pollutant scenarios: 96% of days classified as “unhealthy” by AQI were reclassified as “very unhealthy” or “hazardous” under AAQI. Among days categorized as “very unhealthy” by AQI, 67% and 75% were upgraded to “hazardous” by AAQI and HAQI, respectively^18^. Cheng et al. studied the air pollution status and characteristics of related health risks in 14 cities in Gansu Province. The results showed that from 2020 to 2022, all cities in Gansu were classified as “healthy air quality” according to the EHAQI standards; while according to the AAOI standards, cities in the northwest were classified as “light pollution”^31^. These discrepancies underscored the limitations of single-pollutant metrics in multi-pollutant environments. In our study, AAQI and HAQI exhibited consistent pollution-health correlations, with higher AAQI values corresponding to relatively elevated HAQI levels (though HAQI remained consistently lower than AAQI). However, inter-group variations emerged: the divergence between AAQI and HAQI narrowed in high-pollution areas but expanded in low-pollution zones. This phenomenon was also observed in another similar study, which might be related to the differences in the calculation methods of the indices^18^.

In our study, PM-attributed to ER accounted for nearly 80%, with PM_10_ being the primary contributor. This finding aligned with the previous studies, confirming the significant health risks associated with PM_10_ exposure. A multinational study conducted by Liu et al. showed that each 10 µg/m³ increment in the 2-day moving average concentration of PM10 was associated with a 0.44% increase in the risk of all-cause mortality. Regarding NO₂, although its concentration in Northern Xinjiang was only half that of PM, its ER exceeded that of PM_10_. The oxidizing properties of NO_2_ and O_3_ might act synergistically with arid conditions, thereby significantly amplifying the health risks of diseases^26^. Such pattern differences indicated that health risks were associated not merely with pollution levels, but also closely with emission sources (natural and anthropogenic), social factors (urbanization and population density), and geographical and climatic factors (topography and meteorological conditions)^23–25^. Those contributed to spatial heterogeneity and exposure inequality together^25^. In Southern Xinjiang, local dust sources (the Taklamakan Desert), agricultural activities (e.g., straw and coal combustion) and adverse dispersion conditions (topographic obstruction), contributed to the predominance of health threats from PM₁₀^54,55^. In contrast, Northern Xinjiang was characterized with higher-level industrialization, intensive urban emissions, and complex topography (e.g., the Tianshan Mountains), which favored the formation and accumulation of NO₂ and ozone, leading to NO₂-dominated health risks^56,57^. In spring, dust storms were the most severe, and prolonged synergistic effects of mixed pollutants led to longer population exposure and higher EHAQI in Xinjiang^58^. While in winter, stable atmospheric conditions (e.g., temperature inversions) trapped combustion pollutants near the surface^55,59^. Although Hotan exhibited higher ER and EHAQI values, mortality estimates for Kashgar surpassed Hotan due to its large population base (nearly 4.5 million, the highest in Xinjiang)^5^. Therefore, it was necessary to use population-weighted indices to assess health risks instead of average pollutant concentrations. Targeted interventions for PM₁₀ in Southern Xinjiang and NO₂/O₃ in Northern Xinjiang were necessary, with priority to population-based risk modeling and seasonal interventions for mitigating adverse health effects.

The pathogenesis of air pollutants involves complex mechanisms through which their physicochemical properties interacted with biological systems, acting via distinct pathways. NO₂ was shown to induce airway inflammation and synergistic effects with O_3_^22^. Ozone was associated with oxidative stress in the pulmonary system and the release of systemic cytokines^60^; SO₂ induced bronchial constriction and impaired mitochondrial function in cardiac muscle tissues^61^; CO impaired oxygen transport and brought about neurotoxic lipid peroxidation^62^., and VOCs were recognized as respiratory irritants and carcinogens, while also contributing to O_3_ formation^63^. PM induced more common and complex human damage. PM_2.5_ could penetrate deep into the lungs and bloodstream, and PM_10_—predominant in arid, dust-prone regions—exhibited distinct risks^64^. PM_10_ was primarily deposited in the upper respiratory tract, where it was shown to trigger epithelial irritation and macrophage activation, although its 2.5–10 μm fraction was still capable of affecting bronchioles and exacerbating chronic obstructive pulmonary disease (COPD)^65^. Mineral components in Southern Xinjiang’s dust (e.g., montmorillonite) were shown to generate reactive oxygen species (ROS) through heterogeneous reactions, inducing lipid peroxidation and NF-κB-mediated inflammation^66^. PM_10_ in the tracheobronchial region was found to cause neutrophilic infiltration and fibrosis, with synergistic interactions increasing particle toxicity and DNA damage risks^67^. The unique physicochemical properties and regional sources of PM_10_ established it as a primary driver of respiratory and cardiovascular burdens in arid regions, necessitating targeted mitigation strategies.

Several limitations were identified in this study. Firstly, air quality monitoring stations were sparsely distributed across regions in Xinjiang (approximately 2–3 stations per region), and most were located near urban areas. This likely led to insufficient representativeness of pollutant data and underestimation of associated health risks in rural and desert areas^68,69^. Secondly, although the study concluded that PM_10_ was the dominant pollutant in Xinjiang, further analysis of its sources and causes were not conducted and needs to be pursued in future research^70^. Thirdly, the Period-two was significantly influenced by policy interventions and external factors (e.g., desert edge control projects and the COVID-19 pandemic), whose impacts could not be isolated or excluded from the analysis^71,72^. Finally, the exposure-response coefficient (β) was derived from a national meta-analysis. Xinjiang varied considerably from other regions in pollution profiles, climate, and population vulnerability. The estimated ER and mortality burden were thus approximate and biased, requiring in-depth medical cooperation and algorithm innovation to address its regional applicability.

## Conclusion

This study analyzed the spatiotemporal variations of air pollutants and related health risks in Xinjiang from 2015 to 2024. The results showed that PM₁₀ was the primary pollutant, especially in Southern Xinjiang. Seasonal variations highlighted the high risks posed by spring sandstorms and pollution stagnation caused by stable atmospheric conditions in winter. PM₁₀ and NO₂ were key contributors to health burdens, with regional disparities determined by emission sources and population density (e.g., Kashgar vs. Hotan). AAQI and EHAQI in Southern Xinjiang were higher than those in Northern Xinjiang. These findings could provide scientific evidence for formulating tailored environmental policies and seasonal prevention measures.

Practical strategies to achieve this include: Firstly, promoting clean energy such as natural gas and new energy vehicles, driving technological innovation and upgrading can reduce industrial exhaust emissions (such as SO₂, NOₓ and VOCs)^73^. Secondly, continuing to advance the closure project of the green sand-blocking belt surrounding the Taklamakan Desert and strengthening afforestation publicity, It is highly significant for reducing PM emissions in Xinjiang, especially Southern Xinjiang^74^. Finally, implementing seasonally differentiated exposure mitigation measures is critical: during the high-incidence periods of sandstorms in spring and autumn, issuing sand-dust disaster warnings in a timely manner by meteorological departments and simultaneously improving the emergency response capacity of medical institutions to ensure timely treatment^75^. The government may institute a sandstorm leave by reference to the high-temperature leave model to reduce residents’ exposure risk to air pollution.

## Supplementary Information

Below is the link to the electronic supplementary material.

Supplementary Material 1

## Data Availability

The datasets used and/or analysed during the current study available from the corresponding author on reasonable request **.**.
