# Supplementary material for: Health risk assessment of air pollution in Xinjiang, Northwest China
Source: Sci Rep. 2026 Feb 9;16:7847. doi: 10.1038/s41598-026-39776-x (PMC12953590; doi:10.1038/s41598-026-39776-x)
Supplement: Supplementary file 1 — Supplementary Material 1 [file 41598_2026_39776_MOESM1_ESM.docx]

| Regions | | AAQI/AQI (Mean±SD) | |  |
| --- | --- | --- | --- | --- |
|  |  | ρ=2 | ρ=2.5 | ρ=3 |
| N-X | ALT | 1.38±0.24 | 1.23±0.17 | 1.16±0.13 |
|  | BTL | 1.46±0.16 | 1.29±0.12 | 1.20±0.10 |
|  | CJ | 1.51±0.18 | 1.33±0.13 | 1.23±0.11 |
|  | HM | 1.43±0.16 | 1.27±0.12 | 1.19±0.10 |
|  | TC | 1.48±0.19 | 1.31±0.14 | 1.22±0.11 |
|  | TP | 1.43±0.15 | 1.27±0.11 | 1.19±0.09 |
|  | UR | 1.49±0.16 | 1.32±0.12 | 1.22±0.10 |
|  | IL | 1.58±0.18 | 1.38±0.14 | 1.27±0.11 |
| S-X | AKS | 1.38±0.15 | 1.24±0.11 | 1.17±0.09 |
|  | BY | 1.36±0.16 | 1.22±0.12 | 1.15±0.09 |
|  | HT | 1.31±0.17 | 1.19±0.12 | 1.13±0.09 |
|  | KS | 1.36±0.13 | 1.23±0.09 | 1.16±0.07 |
|  | KZ | 1.33±0.15 | 1.20±0.11 | 1.14±0.08 |

Table S1. Sensitivity analysis of the mean and standard deviation of the AAQI/AQI ratio when choosing ρ values of 2, 2.5, and 3.

Table S2. Statistics on inter-annual mean values of different pollutants in Xinjiang under different time periods (N-X: Northern Xinjiang, S-X: Southern Xinjiang;

P-1: Period-one, P-2: Period-two)

| Regions | | PM_2.5_ (μg/m^3^) | | PM_10_ (μg/m^3^) | | SO_2_ (μg/m^3^) | | NO_2_ (μg/m^3^) | | CO (mg/m^3^) | | O_3_ (μg/m^3^) | |
| --- | --- | --- | --- | --- | --- | --- | --- | --- | --- | --- | --- | --- | --- |
|  |  | P-1 | P-2 | P-1 | P-2 | P-1 | P-2 | P-1 | P-2 | P-1 | P-2 | P-1 | P-2 |
| N-X | ALT | 12.0 | 9.0 | 22.6 | 22.0 | 13.1 | 4.1 | 18.0 | 14.2 | 0.8 | 0.4 | 65.8 | 66.2 |
|  | BTL | 31.7 | 24.0 | 70.4 | 60.2 | 16.9 | 9.7 | 22.2 | 17.7 | 1.0 | 0.5 | 51.2 | 63.5 |
|  | CJ | 57.3 | 49.1 | 103.4 | 85.9 | 15.1 | 8.2 | 42.4 | 32.5 | 1.2 | 0.9 | 50.4 | 61.8 |
|  | HM | 33.0 | 29.4 | 95.2 | 101.1 | 8.7 | 7.8 | 24.6 | 25.7 | 1.1 | 0.6 | 53.6 | 67.8 |
|  | TC | 15.7 | 13.2 | 41.3 | 39.7 | 6.5 | 4.6 | 13.6 | 9.6 | 1.1 | 0.4 | 69.3 | 56.6 |
|  | TP | 63.5 | 48.6 | 163.0 | 146.6 | 14.0 | 7.5 | 37.1 | 30.3 | 1.5 | 1.0 | 56.5 | 71.3 |
|  | UR | 62.0 | 40.8 | 112.6 | 75.3 | 12.5 | 6.8 | 47.8 | 34.0 | 1.3 | 0.8 | 46.6 | 62.4 |
|  | IL | 44.6 | 36.3 | 74.7 | 63.1 | 20.1 | 10.6 | 33.8 | 28.9 | 1.7 | 1.2 | 53.6 | 58.5 |
|  | Mean | 40.0 | 31.3 | 85.4 | 74.2 | 13.4 | 7.4 | 29.9 | 24.1 | 1.2 | 0.7 | 55.9 | 63.5 |
| S-X | AKS | 73.9 | 55.3 | 218.1 | 180.1 | 12.0 | 6.7 | 32.4 | 27.8 | 1.0 | 0.8 | 69.3 | 65.9 |
|  | BY | 49.0 | 41.0 | 165.7 | 152.2 | 7.3 | 4.8 | 25.9 | 22.5 | 1.1 | 0.4 | 66.6 | 71.7 |
|  | HT | 104.7 | 98.9 | 342.8 | 364.0 | 38.0 | 10.8 | 27.0 | 22.4 | 1.3 | 1.0 | 62.1 | 72.1 |
|  | KS | 112.1 | 75.2 | 308.4 | 228.8 | 12.5 | 6.4 | 34.5 | 32.7 | 1.3 | 1.0 | 63.5 | 65.3 |
|  | KZ | 57.6 | 53.5 | 207.9 | 175.9 | 5.9 | 6.8 | 14.0 | 13.9 | 1.0 | 0.6 | 82.4 | 85.8 |
|  | Mean | 79.5 | 64.8 | 248.6 | 220.2 | 15.1 | 7.1 | 26.8 | 23.9 | 1.1 | 0.8 | 68.8 | 72.2 |
| All | Mean | 55.2 | 44.2 | 148.2 | 130.4 | 14.0 | 7.3 | 28.7 | 24.0 | 1.2 | 0.7 | 60.8 | 66.8 |
| CAAQS Grade II | | 35.0 | | 75.0 | | 60.0 | | 40.0 | | 4.0 | | 160.0 | |
